# Posterior Amorphous Corneal Dystrophy Is Associated with a Deletion of Small Leucine-rich Proteoglycans on Chromosome 12

**DOI:** 10.1371/journal.pone.0095037

**Published:** 2014-04-23

**Authors:** Michelle J. Kim, Ricardo F. Frausto, George O. D. Rosenwasser, Tina Bui, Derek J. Le, Edwin M. Stone, Anthony J. Aldave

**Affiliations:** 1 Stein Eye Institute, David Geffen School of Medicine at UCLA, Los Angeles, California, United States of America; 2 The Central Pennsylvania Eye Institute, Hershey, Pennsylvania, United States of America; 3 Department of Ophthalmology, The University of Iowa Hospitals and Clinics, Iowa City, Iowa, United States of America; 4 Howard Hughes Medical Institute, Chevy Chase, Maryland, United States of America; Innsbruck Medical University, Austria

## Abstract

Posterior amorphous corneal dystrophy (PACD) is a rare, autosomal dominant disorder affecting the cornea and iris. Next-generation sequencing of the previously identified PACD linkage interval on chromosome 12q21.33 failed to yield a pathogenic mutation. However, array-based copy number analysis and qPCR were used to detect a hemizygous deletion in the PACD linkage interval containing 4 genes encoding small leucine-rich proteoglycans (SLRPs): *KERA*, *LUM*, *DCN*, and *EPYC*. Two other unrelated families with PACD also demonstrated deletion of these SLRPs, which play important roles in collagen fibrillogenesis and matrix assembly. Given that these genes are essential to the maintenance of corneal clarity and the observation that knockout murine models display corneal phenotypic similarities to PACD, we provide convincing evidence that PACD is associated with haploinsufficiency of these SLRPs.

## Introduction

Posterior amorphous corneal dystrophy (PACD [MIM 612868]) is a rare, autosomal dominant disorder in which affected individuals typically demonstrate partial or complete posterior lamellar corneal opacification, decreased corneal thickness, and flattening of the corneal curvature. Significant phenotypic variability exists, however, not only in the severity of these corneal findings, but also in the expression of a number of other associated abnormalities of the anterior ocular segment, including scleralization of the peripheral cornea, iris coloboma, correctopia, iris atrophy, and iridocorneal adhesions [1]–[10]. Genome-wide linkage analysis performed on the largest PACD family identified to date demonstrated linkage to a 3.5 Mb region on chromosome 12q21.33, to which 26 genes have been mapped (Annotation Release 104) [1]. Four of these genes, keratocan (*KERA* [MIM 603288]), lumican (*LUM* [MIM 600616]), decorin (*DCN* [MIM 125255]), and epiphycan (*EPYC*, also known as *DSPG3* [MIM 601657]), encode small leucine-rich proteoglycans (SLRPs), a family of proteins involved in collagen fibrillogenesis and matrix assembly. In the cornea, these SLRPs are bound to glycosaminoglycans, forming the ground substance of the cornea, and thus play an essential role in the maintenance of corneal transparency [11], [12]. Mutations in the SLRPs have been associated with abnormalities of corneal clarity and curvature, with missense and nonsense mutations in *KERA* implicated in autosomal recessive cornea plana (CNA2 [MIM 217300]) [13]–[20], and nonsense mutations in *DCN* involved in congenital hereditary stromal dystrophy (CSCD [MIM 610048]) [21]–[23]. However, Sanger sequencing of the coding regions of *KERA*, *LUM*, *DCN*, and *EPYC* in the PACD family mapped to chromosome 12q21.33 has failed to identify a pathogenic mutation [1]. After large scale sequencing of the PACD interval also failed to reveal a pathogenic coding region mutation, we performed copy number analysis in this and two other affected families and discovered that PACD is associated with a deletion of *KERA*, *LUM*, *DCN*, and *EPYC*.

## Materials and Methods

### Ethics statement

The researchers followed the tenets of the Declaration of Helsinki in the treatment of the subjects reported in this study, approval for which was obtained from the Institutional Review Board at the University of California at Los Angeles (UCLA IRB 94 – 07-243–23 and 94 – 07- 243–24). All subjects provided written informed consent in accordance with IRB regulations.

### Patient identification and DNA collection

**Figure 1:**
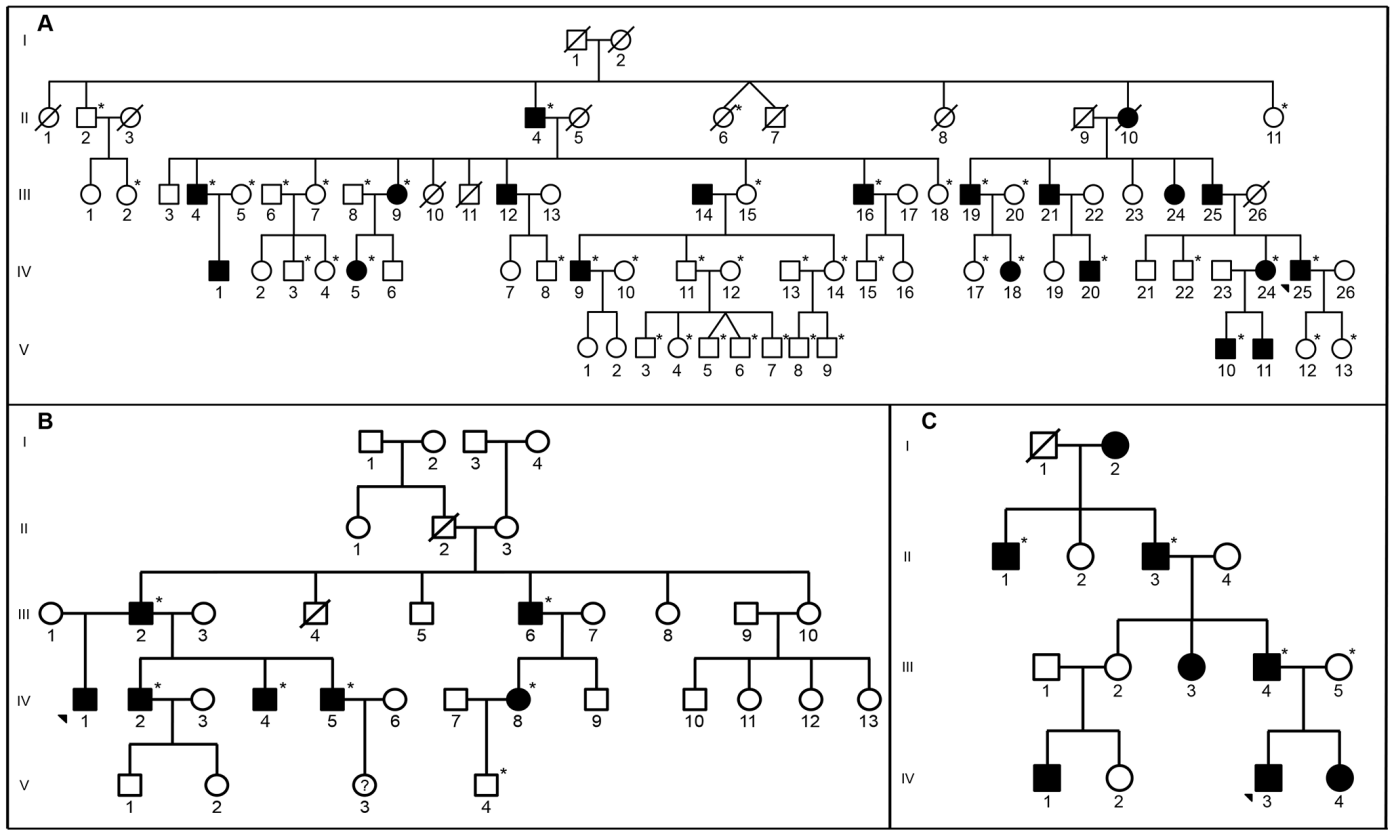
Pedigrees of 3 families with PACD. **A**. Family 1 (abbreviated). **B**. Family 2. **C**. Family 3. Families 1[1] and 3[7] have been reported previously. Asterisks indicate individuals in whom genetic testing was performed. Filled symbols represent affected individuals; open symbols represent unaffected individuals; ? represents individuals of undetermined affected status; arrowhead indicates proband.

Affected and unaffected individuals from 3 families with PACD were enrolled into this study. The clinical criteria used to define the affected phenotype have been previously described [1]. Family 1 was previously used for mapping of PACD to chromosome 12q21.33 [1]. DNA was collected from 2 additional family members (Figure 1A; IV-8 and IV-20), and re-examination of 2 previously recruited individuals (III-9 and IV-5) resulted in the reassignment of their status from unaffected to affected. Family 2 is a previously unreported family in which the clinical features of PACD were identified in 7 individuals, 6 of whom (in addition to 1 unaffected individual) consented to DNA collection (Figure 1B). Family 3 is the family reported by Johnson and colleagues in which the histopathologic features of PACD were first described [7]. Previously collected DNA samples from 4 individuals (3 affected and 1 unaffected) were also included in this study (Figure 1C).

### Next-generation sequencing (NGS) and data analysis

DNA samples from 5 affected (II-4, III-4, IV-18, IV-24, and V-10) and 1 unaffected (IV-17) individual from Family 1 were used for whole-exome sequencing. Genomic DNA was isolated and purified from peripheral blood using the FlexiGene DNA Isolation Kit (Qiagen, Valencia, CA) and from saliva using the Oragene Saliva Collection Kit (DNA Genotek, Inc., Ontario, Canada) and the prepIT•L2P DNA extraction kit (DNA Genotek). After submission to the UCLA Clinical Microarray Core, genomic DNA samples were used to prepare a library using the TruSeq DNA Sample Preparation Kit v2 (Illumina, Inc., San Diego, CA). Whole exome capture was performed using the SeqCap EZ Exome Library v3.0 (Roche NimbleGen, Inc., Madison, WI). Analyses were performed using the default settings for Bowtie2 and SAMtools in the Partek Flow software (Partek, St. Louis, MO). Alignment of the paired-end read sequences to the Human hg19 reference sequence was performed using the Bowtie2 algorithm, while variant discovery (single nucleotide variations (SNV) and insertion-deletions (indels)) was performed using SAMtools mpileup. The bam and bcf files for each sample were imported into the Partek Genomic Suite software for downstream analysis.

### Copy number analysis

Genomic CNV analysis was performed in the UCLA Clinical Microarray Core using the CytoScan® HD Array (Affymetrix, Santa Clara, CA), a high-resolution, genome-wide copy number analytical array containing over 2.6 million probes. Data analysis was performed using the Copy Number Variation module in the Partek Genomics Suite software. Nine samples from Family 1 (II-4, II-6, III-2, III-4, III-18, IV-15, IV-17, V-6, and V-7), 3 samples from Family 2 (III-2, IV-2, and V-4), and 1 sample from Family 3 (IV-3) were submitted for genomic CNV analysis.

### Quantitative PCR (qPCR) and data analysis

Primers to *EPYC* and *DCN*, the most 5′ and 3′ of the 4 SLRPs in the PACD locus, respectively, were created using Primer3 (http://primer3.sourceforge.net). qPCR was performed using the KAPA SYBR FAST qPCR Kit (KAPA Biosystems, Woburn, MA) according to the manufacturer's recommended protocol. Reactions were performed using 10 ng/uL and 1 ng/uL dilutions of genomic DNA. Each sample was run in quadruplicates on the same plate and was repeated on a separate plate to ensure a sufficient number of data points. Samples from a related, unaffected individual (Family 1, IV-17) and an unrelated, unaffected individual were run on each plate to serve as controls. Thermal cycling was performed on the Mastercycler ep realplex (Eppendorf, Hauppauge, NY). Samples were incubated at 95°C for 20 seconds, and then cycled 40 times at 95°C for 3 seconds, 60°C for 20 seconds, and 72°C for 8 seconds. The copy numbers of each sample were calculated by comparing the cycle threshold (Ct) against the two controls. The anti-log of the ΔCt was taken and rescaled to 2 for a diploid locus; a copy number of less than 1.414 was considered to be a heterozygous deletion and a copy number between 1.414 and 2.449 was considered normal [24].

### Family relatedness analysis

Family relatedness was calculated using Mendel (UCLA, Los Angeles, CA), a statistical genetics software package. The Kinship Analysis Option was used to calculate global kinship coefficients, a measure of relatedness, for all possible pairs of genotyped individuals. The calculation used a method-of-moments approach and all observed autosomal genotypes to give an estimate for the overall degree of relatedness for each pair of individuals ranging from a minimum of 0, indicating no relatedness, to a maximum of 0.5, representing the relatedness of an individual to himself or herself [25]. Genotypes were generated using 749,157 genome-wide single nucleotide polymorphisms (SNPs) called by the CytoScan® HD Array from 6 unaffected (II-6, III-2, IV-15, IV-17, V-6, and V-7) individuals from Family 1, 2 affected (III-2 and IV-2) and 1 unaffected (V-4) individuals from Family 2, and 1 affected (III-4) individual from Family 3. The 22,104 X-linked SNPs were not included in the analyses, and an additional 85,853 SNPs were removed due to low genotyping success rates (<98%) and low base call quality (>0.0001) as determined by Chromosome Analysis Suite v2.0 (Affymetrix, Santa Clara, CA). The remaining 641,200 SNPs were available for the analyses.

### Corneal expression of SLRP genes

#### Total RNA isolation and complimentary DNA synthesis

Stroma from donor eye bank corneas denuded of epithelium and endothelium was homogenized in TriReagent (Life Technologies, Carlsbad, CA), and total RNA from the stromal keratocytes and stripped endothelium was isolated separately. Preparation of poly(A)^+^ complimentary DNA from total RNA was performed using oligo(dT)_20_ primers using the protocol for the SuperScript® III First Strand Synthesis System (Life Technologies, Carlsbad, CA).

#### Quantitative polymerase chain reaction (PCR)

Quantitative PCR was performed using transcript-specific oligonucleotides that were obtained from the Harvard Primer Bank database [26]-[28] (http://pga.mgh.harvard.edu/primerbank/index.html) to determine the transcript levels for the SLRP genes (*LUM*, *KERA*, *EPYC* and *DCN*) and coiled-coil glutamate-rich protein 1 (*CCER1* [MIM unavailable]; also known as *C12orf12*; Table 1). *CD34* [MIM 142230] and *VIM* [MIM 193060] transcripts were used as positive controls for stromal keratocytes. Quantitative PCR was performed using 2× KAPA SYBR FAST qPCR Master Mix (KAPA Biosystems, Boston, MA). Reactions were placed in an Eppendorf Mastercycler RealPlex qPCR System (Eppendorf, Hauppauge, NY) with the following reaction conditions: 20 seconds at 95°C, followed by 40 cycles of 3 seconds at 95°C, 20 seconds at 60°C, and 8 seconds at 72°C. Melting curves were determined by one cycle of 15 seconds at 95°C, 15 seconds at 60°C, 20 min ramp time from 60°C to 95°C, followed by 15 seconds at 95°C. Reaction efficiencies (E) were calculated for each transcript-specific oligonucleotide pair using the classical formula E = 10^(−1/slope)^. The oligonucleotides for the SLRP genes and *CCER1* demonstrated similar reaction efficiencies (data not shown). Thus, expression levels of these genes were subsequently analyzed together. Relative expression was obtained by comparison to the housekeeping gene *GAPDH* and calculated using the comparative C_T_ (2^−DDC^T) method. Relative expression levels were plotted as 2^−DC^T values.

**Table 1:** Quantitative PCR oligonucleotides.

Gene	Primer Bank ID	Forward (5′-3′)	Reverse (5′-3′)
SLRPs and *CCER1*			
*CCER1*	34303946c1	CAATAGACCGCACCGATATAGC	TACACCCGAAACACTTGCACC
*EPYC*	223941903c3	CCCAGAAAATCTACGAGCCCT	ACATTGCAGAACGTATCTTCGTG
*LUM*	61742794c3	CTGCGTTTATCTCACAACGAACT	CAGATCCAGCTCAACCAGGG
*KERA*	62865891c3	ACTCACAAAGGTTCCCCGAAT	GGCTGGGACATATTACAGAGACA
*DCN*	47419922c1	ATGAAGGCCACTATCATCCTCC	GTCGCGGTCATCAGGAACTT
Corneal Keratocyte Markers			
*CD34*	68342037c3	ACCAGAGCTATTCCCAAAAGACC	TGCGGCGATTCATCAGGAAAT
*VIM*	240849334c2	AGTCCACTGAGTACCGGAGAC	CATTTCACGCATCTGGCGTTC

#### Statistical analysis

The two-tailed unpaired t-test was utilized for identifying the mean difference in the relative expression levels of *CD34* and *VIM* between corneal stroma and corneal endothelium. One-way analysis of variance (ANOVA) was used to examine the overall mean difference in the expression pattern among all SLRP genes and *CCER1*, and the mean difference of the expression between any two genes was further assessed with Bonferroni correction of multiple comparisons. The differences were considered as statistically significant if p<0.05.

## Results

### Clinical characteristics

#### Family 1

The clinical features of individuals in Family 1 have been described in detail in a previous publication [1]. An additional affected individual that was not included in the previous report (Figure 1A; IV-20) was newly recruited and demonstrated characteristic peripheral, posterior stromal lamellar opacification and decreased central corneal thickness of 443 microns OD and 418 microns OS.

#### Family 2

**Figure 2:**
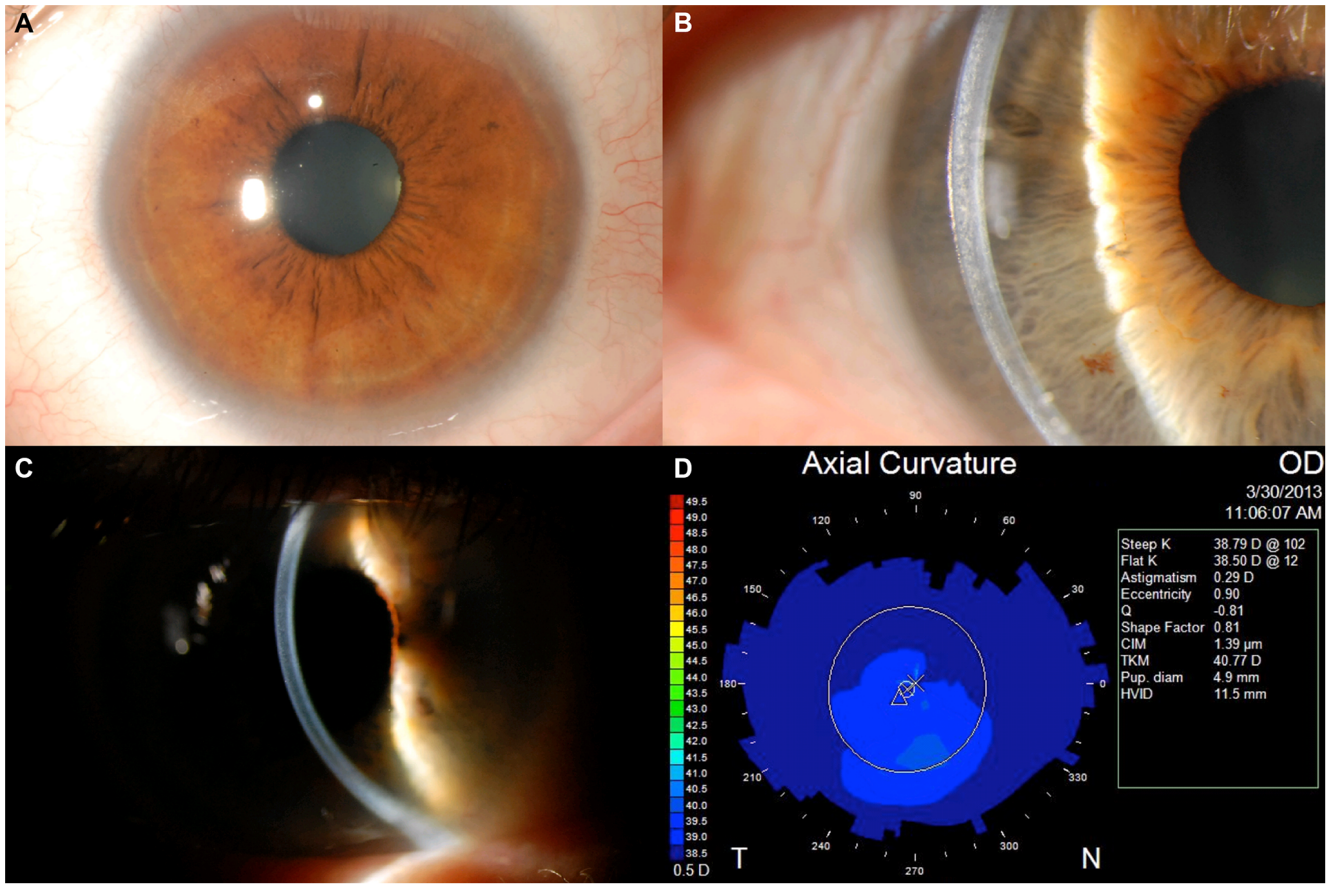
Clinical findings from Family 2. **A**. Slit lamp photomicrograph demonstrating peripheral corneal opacification in individual IV-5. **B**–**C**. Slit lamp photomicrograph of central and peripheral corneal opacification in individuals III-6 (B) and III-2 (C). **D**. Corneal topographic imaging demonstrates significant flattening of the corneal curvature, with a steep K value of 38.79 D, in individual III-2.

Seven individuals in Family 2 were diagnosed as affected with PACD based on the presence of posterior stromal lamellar opacification and decreased central corneal thickness in each eye. In 4 of the affected individuals (Figure 1B; III-2, III-6, IV-1 and IV-2), the posterior lamellar opacification involved the central and peripheral corneal regions, while in 3 of the affected individuals (IV-4, IV-5 and IV-8), the posterior lamellar opacification involved only the peripheral cornea (Figure 2A–C). Central corneal thickness measurements obtained in 6 of the 7 affected individuals (not obtained in IV-1) were less than 500 microns in each eye (individual III-6 is monocular), with the exception of the left eye of IV-4 (503 microns). Corneal topographic imaging was performed in 6 of the 7 affected individuals (not obtained in IV-1), demonstrating significant corneal flattening (average keratometry value <39.0D in each eye) in 2 affected individuals (III-2 and IV-2) and average keratometry values between 42.0D and 45.0D in each eye in the other affected individuals (Figure 2D).

#### Family 3

The clinical features of individuals in Family 3 (Figure 1C) have been described in detail in a previous publication [7].

### Next generation sequencing (NGS)

Three hundred ninety-eight SNVs were identified within the PACD interval in the 5 affected and 1 unaffected individuals from Family 1 whose DNA was analyzed. Four SNVs were present in each of the affected individuals and absent in the unaffected individual, 2 of which were in protein-coding regions but were synonymous substitutions: rs2230282 in *GALNT4* (MIM 603565) and *POC1B-GALNT4* (MIM unavailable) and rs1050395 in *ATP2B1* (MIM 108731). Seventy-four insertions were identified, but only an insertion located in the 3′UTR of *GALNT4* and *POC1B-GALNT4* was present in each of the affected individuals and absent in the unaffected individual. None of the 33 deletions that were identified segregated with the disease phenotype.

### Copy number analysis by cytogenetic array

**Figure 3:**
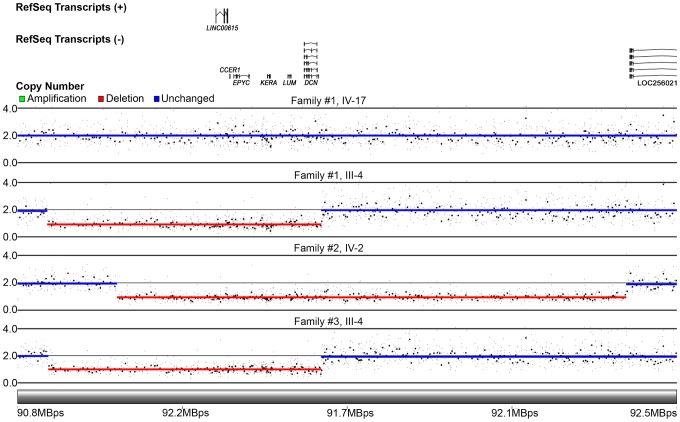
Results of genomic CNV analysis using the CytoScan® HD Array in 3 families with PACD. A schematic of the genes in the region of chromosome 12q21.33 is shown at the top. In Family 1, an unaffected individual (IV-17) without a deletion in the region of chromosome 12q21.33 is shown above an affected individual (III-4) who demonstrates a 701 Kb heterozygous deletion involving *CCER1, KERA*, *LUM*, *DCN*, and *EPYC*. In Family 2, a 1.318 Mb heterozygous deletion involving *CCER1, KERA*, *LUM*, *DCN*, and *EPYC* was identified in an affected individual (IV-2). In Family 3, a 701 Kb heterozygous deletion in chromosome 12q21.33 with identical boundaries to that identified in Family 1 was identified in an affected individual (III-4).

After NGS failed to reveal a presumed pathogenic mutation in the PACD locus, copy number analysis was performed using DNA from 2 affected individuals (Figure 1A; II-4 and III-4) and 2 unaffected individuals (Figure 1A; III-18 and IV-17) in Family 1. The CytoScan® HD Array detected a 701 kb heterozygous deletion in the region of chromosome 12q21.33 (Gene Expression Omnibus accession number GSE50573) containing the four SLRP genes (*EPYC*, *KERA*, *LUM*, and *DCN*), a fifth protein-coding gene, *CCER1*, and a non-protein coding gene (long intergenic non-protein coding RNA 615, *LINC00615* [MIM unavailable]) in both affected individuals that was absent in the unaffected individuals (Figure 3). In Family 2, a 1.318 Mb heterozygous deletion was detected in the 12q21.33 region in 2 affected individuals (Figure 1B; III-2 and IV-2) that was not present in an unaffected individual (Figure 1B; V-4 and Figure 3). Although the deletion was nearly twice as large as that identified in Family 1, the only genes that were involved were the same 6 aforementioned ones. In Family 3, the same 701 kb deletion present in the affected members of Family 1 was identified in an affected individual (Figure 1C; III-4 and Figure 3).

### Copy number analysis by qPCR

**Figure 4:**
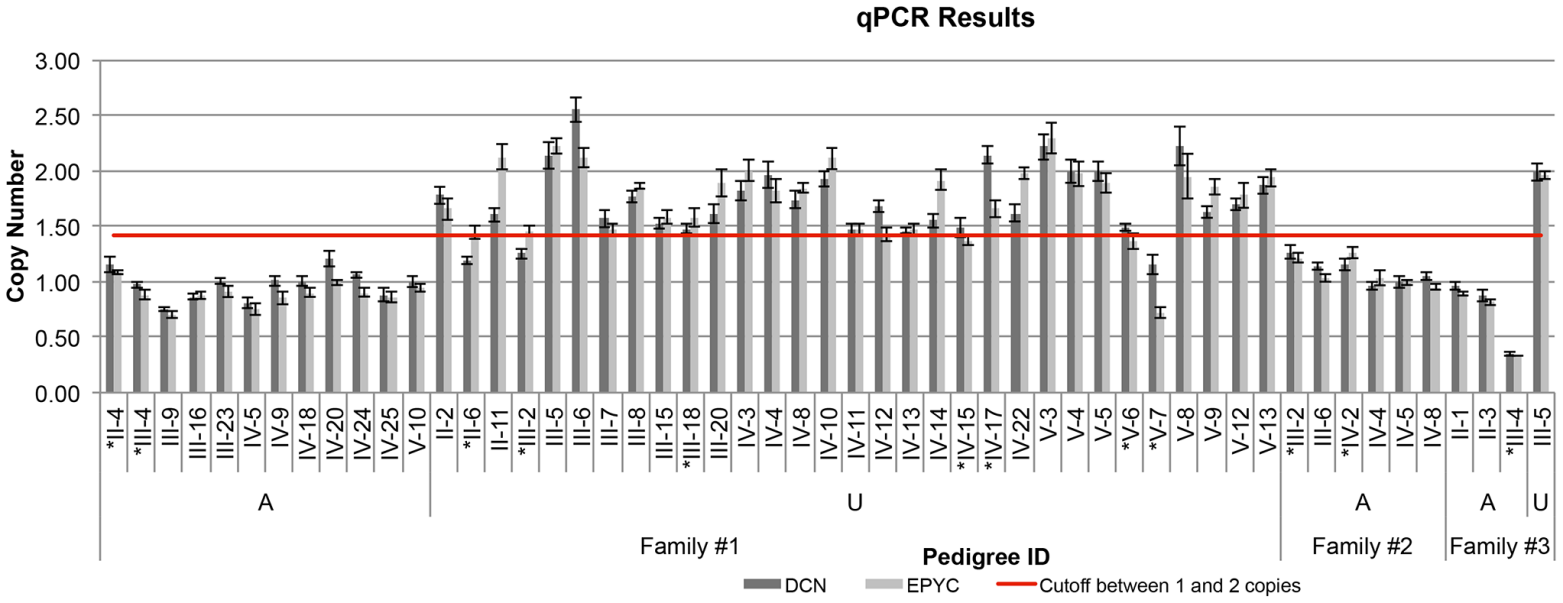
Quantitative PCR results for *EPYC* and *DCN* for 3 families with PACD. The cutoff between 1 and 2 copies is represented by a horizontal red line at 1.414, the geometric mean between 1 and 2. Results are grouped by affected status and arranged in order of location on pedigree. Data are represented as the mean± SEM. A =  affected; U =  unaffected; *Genomic CNV analysis also performed.

To confirm segregation of the deletion in the PACD locus with the affected phenotype in each of the three families, qPCR was performed for all individuals with sufficient DNA samples for copy number analysis. Including the previously mentioned 2 affected and 2 unaffected individuals from Family 1, copy number analysis by qPCR was performed for 43 (12 affected and 31 unaffected) other family members. Quantitative PCR indicated that all 12 affected individuals had only 1 copy of *DCN* and *EPYC* and that 26 of the 31 unaffected individuals had 2 copies of *DCN* and *EPYC* (Figure 4). However, ambiguous results were obtained in 5 individuals: 2 unaffected individuals (Figure 1A; II-6 and III-2) appeared to have 1 copy of *DCN* but 2 copies of *EPYC*; 2 unaffected individuals (Figure 1A; IV-15 and V-6) appeared to have 2 copies of *DCN* but 1 copy of *EPYC*; and 1 unaffected individual (Figure 1A; V-7) appeared to have 1 copy of both *DCN* and *EPYC*. CytoScan® HD Array analysis subsequently performed in these 5 unaffected individuals demonstrated the absence of the deletion identified in the affected individuals.

In Family 2, qPCR was performed for 6 affected and 1 unaffected individuals (including the 2 affected and 1 unaffected individuals in whom CytoScan® HD Array analysis was performed). As in Family 1, all 6 affected individuals were found to have a heterozygous deletion (Figure 4). In Family 3, qPCR performed for 2 affected and 1 unaffected individuals demonstrated a deletion of *DCN* and *EPYC* in the affected individuals that was not present in the unaffected individual. In the affected individual in Family 3 in whom CytoScan® HD Array analysis demonstrated a deletion in the PACD locus (Figure 1C; III-4), the amplified DNA used for qPCR yielded unreliably low copy numbers (Figure 4).

### Family relatedness

To determine whether the 701 kb heterozygous deletion identified in Families 1 and 3 indicated that the mutations and the families descended from a common ancestor, kinship analyses were performed on all possible pairs of 10 genotyped individuals from the 3 families: 6 unaffected individuals from Family 1, 2 affected and 1 unaffected individuals from Family 2, and 1 affected individual from Family 3 (Table S1). All global kinship coefficients between individuals from different families were estimated at 0, indicating that there is no relatedness between each PACD family.

### Corneal expression of SLRP genes

**Figure 5:**
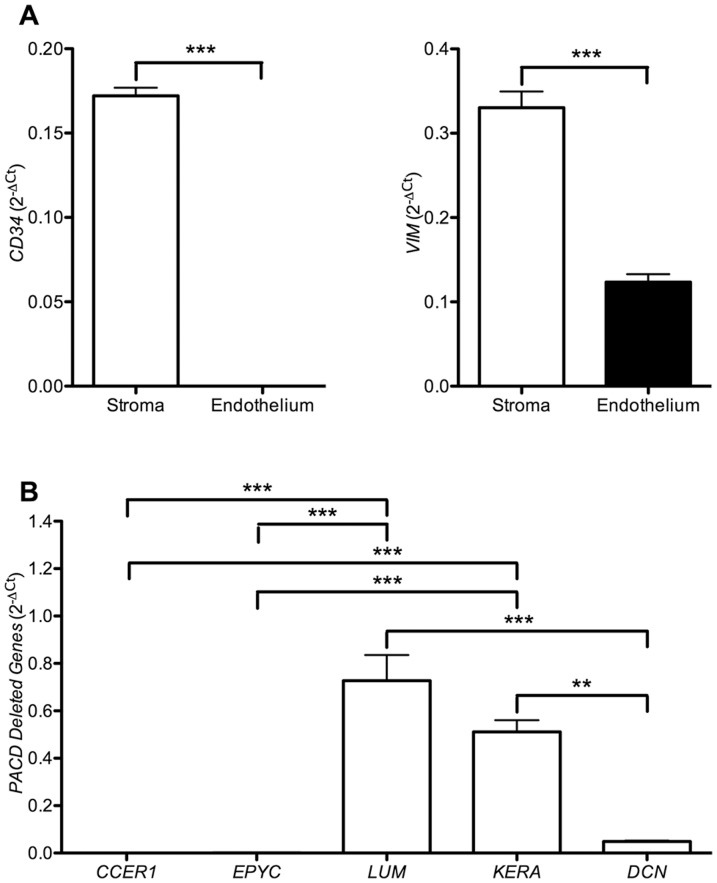
Expression of the SLRP gene cluster and *CCER1* in the corneal stroma by qPCR. **A**. The transcript levels for the keratocyte markers *CD34* and *VIM* were significantly higher in the corneal stroma than in the corneal endothelium. Statistical analysis was performed using a two-tailed unpaired t-test. **B**. Expression of *LUM* and *KERA* was significantly higher than *DCN*, which in turn was higher than the undetectable expression levels of *EPYC* and *CCER1*. Statistical analyses were performed using one-way ANOVA and Bonferroni's Multiple Comparison Test (*p≤0.05; **p≤0.01; ***p≤0.001 (error bars  =  SEM)). Non-significant results are not represented.

Corneal expression of the SLRP genes and *CCER1* was determined by qPCR (Figure 5). Genes known to be preferentially expressed by stromal keratocytes (*CD34* and *VIM*) demonstrated significantly higher expression in the corneal stroma than in the corneal endothelium (Figure 5A). *LUM* and *KERA* exhibited significantly higher expression than *DCN*, although the difference in expression between *LUM* and *KERA* was not significant. While *DCN* demonstrated a higher level of expression than *EPYC* and *CCER1*, the difference was not statistically significant (Figure 5B).

## Discussion

PACD is associated with a heterozygous deletion of the 4 SLRPs, *KERA*, *LUM*, *DCN* and *EPYC*, in the region on chromosome 12q21.33 to which it was previously mapped. The NGS data suggests that it is highly unlikely for a point mutation to be associated with PACD, although we acknowledge that whole-exome sequencing does not always provide sufficiently deep coverage of all coding regions. In this study, however, only regions of low complexity or regions that are highly represented in the genome lacked adequate coverage, making it very unlikely that a pathogenic point mutation was missed. Given this, and the identification of a heterozygous deletion that segregated with the disease phenotype in each of three families, further investigation of these regions would be low-yield.

The only other protein-coding gene contained within the deleted region, *CCER1*, was formerly considered a pseudogene and only recently gained status as a provisional protein-coding gene in the NCBI database. As such, not much is known about its function. However, it is unlikely to play a role in the pathogenesis of PACD as we were unable to detect its expression in the cornea. In contrast, the SLRPs are known to play an essential role in the proper development, structure, and function of the cornea, the transparency of which depends on the SLRP-mediated arrangement of collagen fibrils in a lamellar fashion [11].

Murine models of loss of function of individual SLRPs demonstrate phenotypic features that are consistent with those observed in the 3 PACD families that we report. Keratocan-deficient mice demonstrate decreased corneal stromal thickness and less organized arrangement of stromal collagen fibers, although normal corneal clarity is maintained [29]. Heterozygous lumican-deficient mice also have clear corneas, but posterior corneal stromal opacification is observed in homozygous lumican-deficient mice and is associated with irregular arrangement of thickened collagen fibrils [30]–[32]. As the corneas of lumican-deficient mice demonstrate decreased levels of keratocan expression, and as the expression of lumican and keratocan are directly related, lumican appears to regulate the transcription of *KERA* at the promoter level [33]. As the glycosaminoglycan keratan sulfate associates in the cornea with the proteoglycans lumican and keratocan, loss of lumican expression results in loss of both of the keratan sulfate proteoglycans in the cornea, resulting in decreased corneal stromal thickness and posterior corneal stromal opacification. The converse is not true, however, as the loss of keratocan has been shown not to result in alterations of the expression of any other SLRPs [29]. Ultrastructural examination of the corneas of *Dcn* ^+/−^ and ^−/−^ mice does not demonstrate abnormalities of collagen fibril diameter or organization, and thus alteration of corneal transparency would not be expected [34]. The lack of collagen fibril disorganization in the decorin-deficient mice may be explained by a compensation for loss of *Dcn* expression by biglycan (*BGN* [MIM 301870]), another class I SLRP whose expression is upregulated in decorin-deficient mice [35]. Whereas ultrastructural examination of corneas from *Dcn* and *Bgn*-deficient mice does not demonstrate alterations in collagen fibril structure or arrangement, corneas of double *Dcn*/*Bgn* null mice demonstrate significant alterations in the posterior cornea, indicating that biglycan compensates for a loss of decorin [35].

The role of the fourth deleted SLRP, epiphycan, in PACD remains unclear as its role in the cornea, if any, has not been elucidated. As epiphycan is predominantly expressed in cartilage, reports of examination of epiphycan-deficient mice have focused on the knee joint of the mice and have not involved an assessment of corneal clarity, thickness or contour [36]–[38]. However, as with *CCER1*, our failure to identify expression of *EPYC* in the cornea indicates that it is unlikely to play a role in the pathogenesis of PACD.

As each of the 3 unrelated families with PACD demonstrated a deletion of *EPYC*, *KERA*, *LUM*, and *DCN*, it is not definitively known which gene, or combination of genes, is essential to the pathophysiology of PACD. However, based on the corneal phenotype observed in mice deficient for each of the SLRPs and the aforementioned interaction between the SLRPs expressed in the cornea, *LUM* deficiency is likely necessary and may be sufficient to cause PACD. As noted previously, keratocan-deficient mice do not demonstrate corneal stromal opacification, a sine-qua-non of PACD, and decorin-deficient mice do not demonstrate alterations in collagen fibril structure or arrangement. Additionally, deletion of *DCN* has been identified in 8% of control individuals, indicating that *DCN* deletion alone is not sufficient to cause PACD, which has been identified in only 13 families to date (including this report) [1]–[10], [39]. A deletion involving both *DCN* and *LUM* has been described in 3 of 2493 (0.12%) control individuals, although as ocular examinations were not performed, it is not known whether deletion of these two SLRPs is sufficient to produce the PACD phenotype [40]. Screening of additional families with PACD will likely reveal interstitial deletions of the PACD locus in each, and depending on the extent of the deleted region, may suggest that *LUM* deficiency alone is sufficient to cause PACD or that it is the additive effect of the deletion of several SLRPs that is involved in the pathogenesis of PACD.

Other findings in PACD, such as corneal scleralization, flattening, and iris abnormalities, have not been sufficiently explained by murine models of loss of function of individual SLRPs, as measurements of corneal curvature and descriptions of iris phenotypes were not reported in studies of these knockout mice. *LUM* and *KERA* have been shown to be expressed in iris tissue in mouse and chick models, while *DCN* and *EPYC* have not been reported to be expressed in the iris, to the best of our knowledge [41], [42]. More complete ocular examinations in murine models and the creation of serial knockout mice with various combinations of SLRP deletions would likely determine which are involved in the pathogenesis of PACD.

Autosomal dominant cornea plana (CNA1 [MIM 121400]) is a dysgenesis of the anterior segment of the eye characterized by clinical features associated with PACD such as corneal flattening, corneal stromal haze and pupillary abnormalities, as well as distinct phenotypic features such as microcornea and posterior embryotoxon [43], [44]. Although autosomal dominant cornea plana has been mapped to a 3cM region of chromosome 12q that contains the SLRP gene cluster, a pathogenic coding region mutation has not been identified [43]. A child with a 24.8 Mb interstitial deletion of chromosome 12q15-q23, which contains the SLRP gene cluster and 156 other genes (Annotation Release 104), has been reported to exhibit corneal opacification, microcornea, small and irregular pupils, and central iridocorneal adhesions, consistent with autosomal dominant cornea plana [45]. A possible explanation for the difference in the phenotype observed in this individual and the families with PACD that we report is that the larger deletion contains genes that contribute to the clinical features unique to autosomal dominant cornea plana. Alternatively, the child with the large interstitial deletion may actually have autosomal recessive cornea plana with unmasking of a recessive allele, a possibility that was not excluded as *KERA* was not screened.

CNVs can cause disease through various mechanisms, including gene dosage, gene interruption, gene fusion, position effect resulting in the alteration of regulatory regions, unmasking of recessive alleles, and transvection (*trans* regulation of gene expression mediated by the pairing of alleles on homologous chromosomes) [46]. Of these, a gene dosage effect is the most likely pathogenic mechanism for PACD. In a study of chick corneas, the concentrations of keratan sulfates (like keratocan and lumican) and chondroitin/dermatan sulfates (like decorin) changed during embryonic development along with corneal clarity [47]. The maximal accumulation of keratan sulfates at the end of embryonic development and adulthood correlated with the maximal transparency of the cornea. On the other hand, chondroitin/dermatan sulfates decreased during development and were lowest during adulthood, suggesting that the relative expression of these two types of proteoglycans may regulate corneal clarity [47]. A disruption of the expression of the keratan sulfates, due to a heterozygous deletion involving the SLRPs, could result in a subsequent alteration in the relative expression of the different corneal proteoglycans. As keratocan and lumican exhibit significantly higher corneal expression than decorin and epiphycan, a relative decrease in their expression would be expected to disrupt the role of the glycosaminoglycans in the maintenance of corneal clarity. Other mechanisms via which the identified deletion of chromosome 12q21.33 could cause PACD are less likely, as the boundaries of the deletion are in intergenic regions (making gene interruption, gene fusion and position effects unlikely) and no segregating mutations were identified by NGS, making unmasking of recessive alleles an improbable mechanism.

In summary, the implication of the deletion of the SLRP gene cluster as the genetic basis of PACD provides additional evidence of the role of these genes in the development and maintenance of corneal clarity. Given this important role, the phenotypic similarities in animal models and the demonstration of an overlapping deletion in 3 unrelated families, PACD is associated with, and likely caused by, haploinsufficiency of SLRPs on chromosome 12.

## Supporting Information

Table S1 **Calculated kinship coefficients between individuals from 3 families with PACD.** *Numbers assigned to each individual are those used to designate pedigree position in Figure 1. Bolded kinship coefficients are comparisons between individuals from different families. Dashes indicate duplicate kinship coefficients.
(DOCX)
