# Supplementary material for: Posterior Amorphous Corneal Dystrophy Is Associated with a Deletion of Small Leucine-rich Proteoglycans on Chromosome 12
Source: PLoS One. 2014 Apr 23;9(4):e95037. doi: 10.1371/journal.pone.0095037 (PMC3997350; doi:10.1371/journal.pone.0095037)
Supplement: Table S1 — Calculated kinship coefficients between individuals from 3 families with PACD. *Numbers assigned to each individual are those used to designate pedigree position in Figure 1. Bolded kinship coefficients are comparisons between individuals from different families. Dashes indicate duplicate kinship coefficients. (DOCX) [file pone.0095037.s001.docx]

Table S1

|  |  |  |  | | | | | | | | |
| --- | --- | --- | --- | --- | --- | --- | --- | --- | --- | --- | --- |
|  | | Family 1 (F1) | | | | | | Family 2 (F2) | | | Family 3 (F3) |
| Individual* | | **II-6** | **III-2** | **IV-15** | **IV-17** | **V-6** | **V-7** | **III-2** | **IV-2** | **V-4** | **III-4** |
| F1: **II-6** | | 0.5000 | 0.0487 | 0.0000 | 0.0011 | 0.0000 | 0.0000 | **0.0000** | **0.0000** | **0.0000** | **0.0000** |
| F1: **III-2** | | - | 0.5000 | 0.0000 | 0.0000 | 0.0000 | 0.0000 | **0.0000** | **0.0000** | **0.0000** | **0.0000** |
| F1: **IV-15** | | - | - | 0.5000 | 0.0000 | 0.0000 | 0.0000 | **0.0000** | **0.0000** | **0.0000** | **0.0000** |
| F1: **IV-17** | | - | - | - | 0.5000 | 0.0000 | 0.0000 | **0.0000** | **0.0000** | **0.0000** | **0.0000** |
| F1: **V-6** | | - | - | - | - | 0.5000 | 0.4687 | **0.0000** | **0.0000** | **0.0000** | **0.0000** |
| F1: **V-7** | | - | - | - | - | - | 0.5000 | **0.0000** | **0.0000** | **0.0000** | **0.0000** |
| F2: **III-2** | | - | - | - | - | - | - | 0.5000 | 0.1827 | 0.0000 | **0.0000** |
| F2: **IV-2** | | - | - | - | - | - | - | - | 0.5000 | 0.0000 | **0.0000** |
| F2: **V-4** | | - | - | - | - | - | - | - | - | 0.5000 | **0.0000** |
| F3: **III-4** | | - | - | - | - | - | - | - | - | - | 0.5000 |
